# Role of Vascular Endothelial Cells in Disseminated Intravascular Coagulation Induced by Seawater Immersion in a Rat Trauma Model

**DOI:** 10.1155/2017/5147532

**Published:** 2017-06-28

**Authors:** Dajin Zhang, Jia Qu, Ming Xiong, Yuanyuan Qiao, Dapeng Wang, Fengjiao Liu, Dandan Li, Ming Hu, Jiashu Zhang, Fuyu Wang, Xiaohang Zhao, Chenghe Shi

**Affiliations:** ^1^Center for Basic Medical Sciences, Navy General Hospital of Chinese PLA, Beijing 100048, China; ^2^Department of Neurosurgery, PLA 301 Hospital, Beijing 100853, China

## Abstract

Trauma complicated by seawater immersion is a complex pathophysiological process with higher mortality than trauma occurring on land. This study investigated the role of vascular endothelial cells (VECs) in trauma development in a seawater environment. An open abdominal injury rat model was used. The rat core temperatures in the seawater (SW, 22°C) group and normal sodium (NS, 22°C) group declined equivalently. No rats died within 12 hours in the control and NS groups. However, the median lethal time of the rats in the SW group was only 260 minutes. Among the 84 genes involved in rat VEC biology, the genes exhibiting the high expression changes (84.62%, 11/13) on a qPCR array were associated with thrombin activity. The plasma activated partial thromboplastin time and fibrinogen and vWF levels decreased, whereas the prothrombin time and TFPI levels increased, indicating intrinsic and extrinsic coagulation pathway activation and inhibition, respectively. The plasma plasminogen, FDP, and D-dimer levels were elevated after 2 hours, and those of uPA, tPA, and PAI-1 exhibited marked changes, indicating disseminated intravascular coagulation (DIC). Additionally, multiorgan haemorrhagia was observed. It indicated that seawater immersion during trauma may increase DIC, elevating mortality. VECs injury might play an essential role in this process.

## 1. Introduction

In the wake of the rapid development of the marine economy and communication, yearly disasters at sea are unavoidable globally. Seawater has distinct physicochemical properties. The heat conductivity and specific heat of seawater are much higher than those of air, and thus it is easier for the body to develop hypothermia in seawater than on land [1]. Seawater contains a multiplicity of ions, including approximately 3 times more sodium ions than human plasma, creating much higher osmotic pressure [2]. Such special characteristics lead to the development of a different course for a trauma victim who is exposed to a seawater environment.

Over the past decade, the course of various types of trauma complicated by seawater immersion have been observed in detail, including the analysis of plasma electrolyte levels, blood gas, blood pressure, heart function, and histopathology. The results have revealed serious hyperosmotic anhydration, metabolic acidosis, electrolyte imbalances, microcirculation disturbances, and cell degeneration and necrosis [3–5], making rescue and treatment more complicated and difficult for victims in marine accidents [6, 7]. Furthermore, the mechanisms of compensation and decompensation in the body remain unclear.

Vascular endothelial cells (VECs) are a type of tissue cell widely found in the human body, with special clinical significance in maintaining blood flow and regulating vascular tone and material exchange, as well as preventing platelet aggregation and thrombosis. VECs not only are the protective layer situated between the blood circulation and tissues but also possess metabolic and endocrine functions [8, 9]. Therefore, injury to VECs is tightly linked to the development of many diseases [10, 11].

When a trauma victim has fallen into the sea, the seawater not only may stimulate the wound tissue directly but also may penetrate the wound and enter into the abdominal cavity or thoracic cavity. Ions then dialyse from the cavity to the circulation across the abdominal peritoneal membrane or the pleura due to the extremely high osmotic pressure gradient [12, 13]. The main focuses of this study were the response of VECs, as the front line of defence with respect to the movement of material from blood to tissue and constitute the largest secretory system in the body, to such acute osmotic changes and the effect of endothelial activation or dysfunction on the development of trauma during seawater immersion. An animal trauma model was used. Changes in the expression of functional genes in VECs were detected. The indicated functional disturbance in blood coagulation was then further studied.

## 2. Material and Methods

### 2.1. Open Abdominal Injury Rat Model and Survival Time

**Figure 1:**
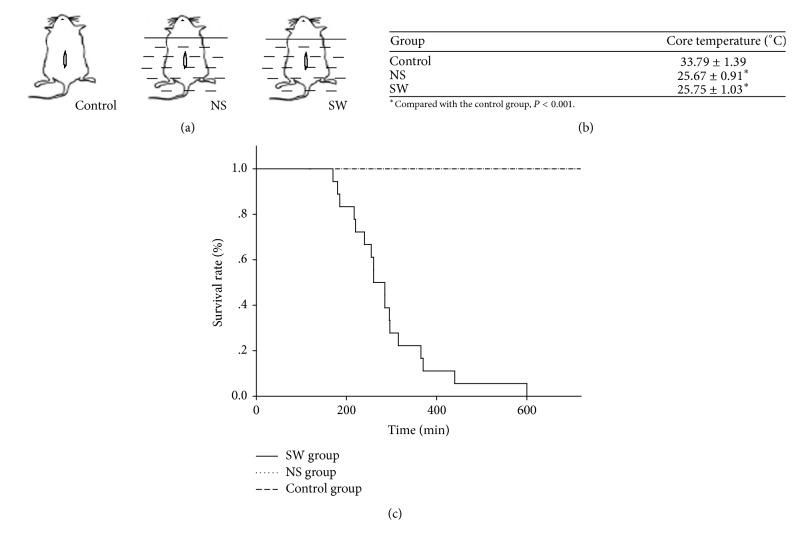
Survival time of rats with an open abdominal injury in different environments. (a) An open abdominal injury animal model was established. Rats in the treatment group were vertically immersed into 22°C seawater (SW group) or normal saline (NS group). Rats in the control group were directly placed in a 22°C environment (control group). (b) The table displays the rectal temperatures of the test rats after 120 minutes of exposure. The rectal temperatures of the test animals were measured at different times. The rectal temperatures of the rats in both the NS group and SW group decreased rapidly but synchronously. (c) No rats in the NS group or control group died in 720 min. However, the average survival time of the rats in the SW group was 276 min, with a median lethal time of 260 minutes.

The animal experimentation protocol was reviewed and approved by the Ethical Committee of the Navy General Hospital. The experimental seawater was prepared according to the standard of the Third Institute of Oceanography of the State Ocean Bureau: osmotic pressure 1250.00 ± 11.52 mOsm/L, pH 8.20, [Na^+^] 630.00 ± 5.33 mmol/L, [K^+^] 10.88 ± 0.68 mmol/L, and [Cl^−^] 658.80 ± 5.25 mmol/L. Wistar male rats weighing 200–220 g were anaesthetized with an intraperitoneal injection of 1% pentobarbital sodium (30 mg/kg body weight). An open abdominal injury was induced with a 3 cm median abdominal incision 2 cm below the cartilago ensiformis, with a plastic perforated sheet to prop open the incision [7]. Rats in the treatment groups were vertically immersed into 22°C seawater (SW) or normal saline (NS), with the water surface aligned to the armpit. Rats in the control group were directly placed in a 22°C environment (Figure 1(a)). The rectal temperature and other vital indicators were observed for 12 hours unless the rat died as judged by respiratory and cardiac arrest. For histological analyses, the heart, kidney, lung, and intestine were fixed with formaldehyde and paraffin embedded, and then 10 *μ*m microsections were stained with haematoxylin and eosin and analysed by light microscopy (Nikon Eclipse Ti-S).

### 2.2. Assessment of the Expression of Functional VEC Genes Using a Real-Time Quantitative PCR Array

The open abdominal injury rat model was established as above. Two hours after the treatment, the vascular tissue of the small intestinal mesentery was harvested and homogenized in 1 ml of TRIzol Reagent (Invitrogen) per 50–100 mg. Total RNA was isolated according to the manufacturer's protocol and was cleaned using the RNeasy MinElute™ Cleanup Kit (Qiagen). After yield and quality assessment, four RNA samples from either the control or SW group were mixed equally to form a sample pool. Two individual RNA samples in each group were also assayed in parallel. For the PCR array experiments, an RT^2^ Profiler™ PCR Array Rat VECs Biology (SuperArray Bioscience) was used to simultaneously examine the mRNA levels of 89 genes, including five housekeeping genes, in 96-well plates according to the manufacturer's protocol. For the data analysis, the ΔΔCt method was used. For each gene, changes that were calculated to be greater than 2-fold were considered an up- or downregulated difference in gene expression between the control and SW groups [14].

### 2.3. Enzyme-Linked Immunosorbent Assay (ELISA)

Blood was collected via the left ventricle of the rats into test tubes containing 3.8% sodium citrate solution (1 volume to 9 volumes of blood) at 0, 0.5, 1, 2, 3, and 4 hours after the operation with or without seawater immersion. The samples were centrifuged at 1,000 ×g for 15 minutes, and the plasma was separated within one hour. To detect the levels of coagulation-related proteins in plasma, urokinase-like plasminogen activator (uPA), tissue-type plasminogen activator (tPA), plasminogen activator inhibitor type-1 (PAI-1), tissue factor pathway inhibitor (TFPI), and von Willebrand factor (vWF) were measured with a double-antibody sandwich ELISA according to the manufacturer's protocol. Briefly, 100 *μ*l of plasma and 50 *μ*l of enzyme conjugate were dispensed into the antibody precoated 96-well ELISA plate and incubated for 1 hour at 37°C. The ELISA plate was washed 5 times with PBS and then incubated for 15 minutes with a TMB substrate. The colour intensity was measured with an ELISA reader at A450.

### 2.4. Blood Coagulation Tests

Blood was collected, and the plasma was separated as above. The prothrombin time (PT), activated partial thromboplastin time (APTT), and levels of fibrinogen (FIB), plasminogen (PLG), fibrin degradation product (FDP), and D-dimer were measured using a coagulometer (CX5; Beckman, USA).

### 2.5. Statistical Analysis

The results were presented as the means ± standard deviation (SD) from 10 animals. The statistical significance of differences between the treatment and control groups or between the two treatments was analysed using the paired *t*-test or *R* × *C* chi-squared test. *P* < 0.05 was considered to indicate a statistically significant difference for all the data analyses.

## 3. Results

### 3.1. Seawater Exposure Shortened the Survival Time of the Rats with an Open Abdominal Injury

To study the effects of exposure to a seawater environment on the survival of a trauma, the survival times of the rats with an open abdominal injury in the SW, NS, and control groups were observed. The survival times of the rats were markedly shortened when the animals were exposed to 22°C seawater. However, the respiration and heart rate of the rats in the NS or control group remained stable, and no rats died within 12 hours. The rats in the SW group began to die at 170 minutes as judged by respiratory and cardiac arrest, with a median lethal time of 260 minutes (Figure 1(c)). To determine whether hypothermia affected the survival rate, the rectal temperatures of the test animals were measured at different times. The rectal temperatures of the rats in both the NS and SW groups declined rapidly but synchronously and stabilized to 25.67 ± 0.91°C and 25.75 ± 1.03°C, respectively, in 2 hours. However, the rectal temperature of the rats in the control group decreased more slowly and slightly (Figure 1(b)). These results indicated that exposure to a liquid environment easily led to hypothermia but was not the key factor that accelerated the death of the test rats in the SW group.

### 3.2. Seawater Exposure Induced Changes in the Expression of Functional VEC Genes

The expression levels of 84 functional genes, which are involved in permeability, tonicity, angiogenesis, endothelial cell activation, and injury, in VECs of the small intestinal mesentery, were detected with a real-time quantitative PCR array. According to the qPCR array results, compared with those of the rats in the control group, the expression levels of 65 functional VEC genes were markedly changed (77.38%, 65/84) in the rats in the SW group, including 52 genes (61.90%, 52/84) that were upregulated and 13 genes (15.48%, 13/84) that were downregulated. Permeability and vessel tone is the gene class that exhibited the most dramatic change (100%, 12/12), followed by angiogenesis genes (93.33%, 14/15). The gene class of VEC injury, composed of genes concerning the response to stress and apoptosis, changed the least (66.67%, 26/39) (Table 1).

**Figure 2:**
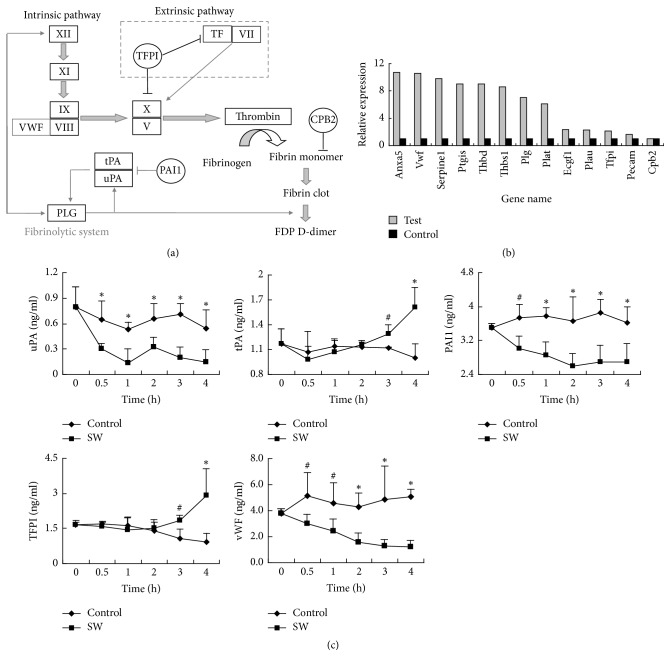
Relationships between functional VEC genes and coagulation cascades. (a) Roles of the functional VEC genes in the coagulation cascades. (b) Compared with the control group, the expression levels of 11 of 13 VEC genes in coagulation cascades in the SW group were increased by more than 2-fold, and 8 of 13 genes changed by more than 6-fold according to the Rat VEC Biology PCR Array. (c) Serum levels of coagulation-related factors in the test rats were assessed with a double-antibody sandwich ELISA. ^#^*P* < 0.05; ^*∗*^*P* < 0.01.

According to the PCR array gene class table, the expression levels of genes concerning thrombin activity were increased significantly (100%, 6/6) (Table 1). Considering all the genes in coagulation cascades (Figure 2(a)), the expression levels of 84.62% (11/13) of the genes were increased by more than 2-fold, with Anxa5, Vwf, Serpine1, Ptgis, Thbd, Thbs1, Plg, and Plat being elevated by more than 6-fold (Figure 2(b)).

### 3.3. Plasma Levels of Proteins Involved in Coagulation Cascades Changed in a Time-Dependent Manner

The levels of proteins involved in coagulation cascades in the blood of the rats of the control and SW groups were examined at different time points. The level of TFPI, a specific inhibitor of the extrinsic coagulation pathway, in the blood of the rats in the SW group was elevated from 2 hours after seawater exposure and was 2.11-fold greater than that in the rats of the control group at 4 hours. The level of vWF in plasma decreased in a time-dependent manner when the rats were exposed to seawater. Although the level of uPA in plasma decreased when the rats were immersed in seawater, the level of tPA increased after 2 hours and was approximately 1.62-fold higher than that of the control group at 4 hours. The level of PAI-1 in plasma apparently decreased in a time-dependent manner (Figure 2(c)). These results suggested that the fibrinolytic system was activated.

### 3.4. Coagulopathy in the Blood of the Rats with Open Abdominal Injury When Exposed to Seawater

**Figure 3:**
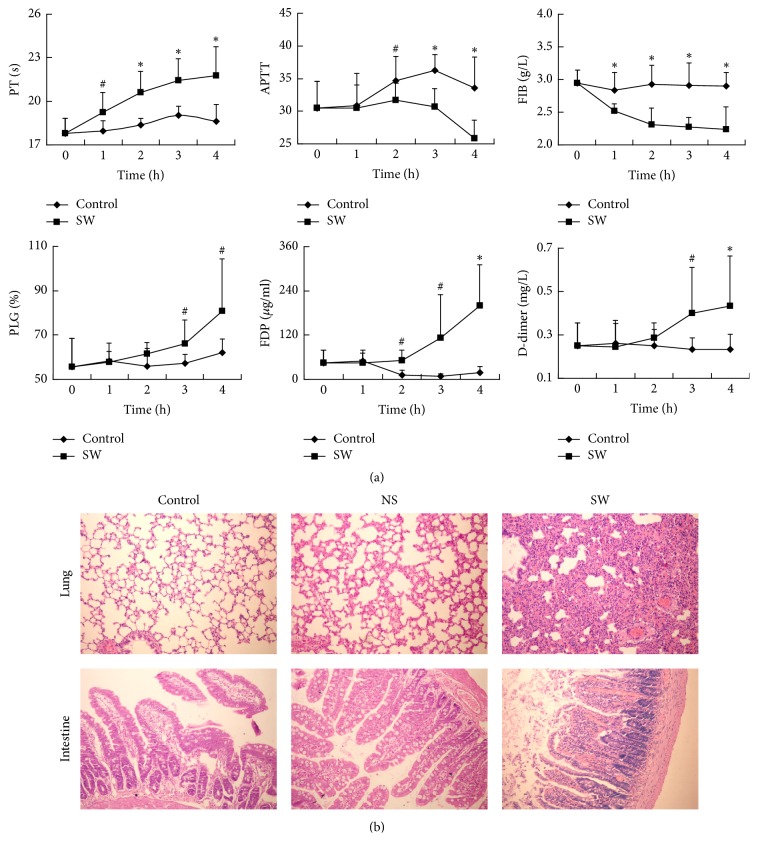
Coagulopathy of rats with an open abdominal injury complicated by seawater immersion. (a) Compared with the control group, the coagulation and DIC-related parameters in the plasma of the rats of the SW group were changed markedly, especially after 2 hours. ^#^*P* < 0.05; ^*∗*^*P* < 0.01. (b) A histological analysis revealed extensive haemorrhaging in multiple organs of the rats in the SW group, especially in the lung and intestine (HE 100x).

The effects of seawater exposure on blood coagulation in the rats with an open abdominal injury were studied at different time periods. Compared with the rats in the control group, the coagulation-related parameters in the rats of the SW group changed markedly. The PT was prolonged in a time-dependent manner, exceeding 3 seconds longer at the 3-hour time point than at the 0-hour time point. The APTT was shortened, and the plasma FIB level was decreased when the rats were exposed to seawater, suggesting that the extrinsic coagulation pathway was inhibited and that the intrinsic coagulation pathway was initiated (Figure 3(a)). These results indicated that seawater immersion activated blood coagulation in the rats with an open abdominal injury.

### 3.5. Disseminated Intravascular Coagulation (DIC) Was Induced during the Late Period of Seawater Exposure

The level of FDP in plasma was elevated after two hours of seawater exposure and reached approximately 200 *µ*g/ml at 4 hours. The levels of PLG and the D-dimer in plasma were increased a little later than those of FDP. However, the three parameters mentioned above did not change appreciably in the rats of the control group (Figure 3(a)). The histological analysis showed extensive haemorrhaging in multiple organs of the rats in the SW group, especially in the lung and intestine (Figure 3(b)). Therefore, during the late period of seawater immersion, the injury model rats tended to develop DIC.

## 4. Discussion

Unfortunately, victims who fall into seawater during disasters at sea tend to have an increased likelihood of death, especially those with an injury. Victims must face loneliness, fear, and a shortage of food and water. Additionally, victims usually develop hypothermia in cold water [15]. In some studies, the authors have suggested that hypothermia is the most important factor affecting the mortality of these victims [16]. In this study, using an animal model, we focused on the role of endothelial injury in inducing DIC in cases of trauma complicated by seawater immersion. DIC may be one of the most important factors leading to the high mortality rate in sea disasters.

Previous reports have indicated that the progression of injury with seawater immersion is dramatically fast and complicated by serious hyperosmotic anhydration, metabolic acidosis, electrolyte imbalance, and microcirculation disturbance, with death occurring within hours [17, 18]. However, further understanding is still lacking regarding the specific fatal factor and the mechanism involved in priming this progression. Different from the air environment over land, three types of physicochemical properties of seawater may have specific effects on human physiology, including the hydrostatic pressure, the high heat conductivity and specific heat, and the multiplicity of high concentration ions.

Under immersion, the hydrostatic pressure lowers the peripheral vascular capacity and increases vascular perfusion. Initial renal and cardiovascular responses are common for both dry immersion and “wet” immersion, with an acute increase in water and electrolyte excretion and changes in haemodynamic parameters [19]. No significant changes in haemostasis system indices were observed in the healthy volunteers during the 7-day dry immersion experiment, with the exception of AP activity, which increased on the 3rd day of the experiment [20]. Endothelium-dependent vasodilation was reduced by dry immersion, and this reduction was accompanied by an increase in circulating endothelial microparticles, which was significant on day 3 [21]. Animals with an open abdominal wound tended to die within a few hours during seawater immersion. According to the results of the dry immersion experiment, short-term microgravity or hydrostatic pressure may not induce the apparent coagulopathy observed in the rats with an open abdominal injury during seawater immersion, but it may affect haemodynamics during the early stage of seawater immersion.

The heat conductivity and specific heat of water are much higher than those of air, and thus, it is easier for the body to develop hypothermia in seawater than on land. In this study, the rectal temperatures of the rats in both the NS and SW groups decreased equivalently, stabilizing to just 3-4°C above the environmental temperature in 2 hours. The temperature of the rats in the control group was 33.79 ± 1.39°C. However, the median lethal time of the rats in the SW group was 260 minutes when exposed to 22°C seawater. No rat in the NS or control group died within 12 hours. It is important to note that “core temperature afterdrop” and “circumrescue collapse” can be caused by the sudden movement or removal of victims from the water following cold-water immersion [15, 22]. Therefore, the rectal temperature and other vital indicator observations were performed very carefully in the rats to avoid death due to man-made disturbances. The results of this study indicate that exposure to a liquid environment easily leads to hypothermia. However, hypothermia is not the key factor that accelerates the death of the rats with an open abdominal injury exposed to seawater immersion.

Differences between the components in seawater and normal saline may determine the fates of the model rats. A high concentration of ions in seawater could penetrate the wound, enter the abdominal cavity, and then be dialysed from the abdominal cavity to the circulation across the peritoneum due to the extremely high osmotic pressure gradient. Reports have shown that the serum sodium and chloride ion concentrations of the animals with an open abdominal injury and exposure to seawater were much higher than those of the control group [23, 24]. Changes in the circulation could not only disturb the electrolyte balance and acid-base equilibrium but also harm the structure or function of the vascular endothelium.

The vascular endothelium is the body's first line of defence against harm transmitted via the circulation [25]. In the past, the endothelium was considered inert, acting as a nonthrombogenic surface for blood flow. However, it is now becoming clear that VECs actively and reactively participate in haemostasis as well as immune and inflammatory reactions. These cells produce and react to various cytokines and adhesion molecules, and it is now evident that VECs can mount anti- and proinflammatory and protective responses depending on the environmental conditions [26, 27]. Endothelial dysfunction or activation contributes to a variety of diseases, such as cancer, rheumatoid arthritis, sepsis, lupus, and psoriasis [28, 29].

To investigate the effects on the endothelium of the components in the seawater permeating from the peritoneal cavity to the circulation via dialysis through the peritoneum, we used a real-time quantitative PCR array and identified 84 functional genes of the rat VECs that were involved in permeability, tonicity, angiogenesis, endothelial cell activation, and injury [14]. Sixty-five genes were differentially regulated over the 2-hour period during which the open abdominal wound was exposed to the seawater. The expression levels of the genes concerning thrombin activity increased significantly.

Normal VECs are inactive against both visible and invisible elements in the blood, forming a thromboprophylactic surface. The structure and functional integrity of VECs are important in the maintenance of the vessel wall and circulatory function [30]. VECs express various proteins that directly participate in coagulation and haemostasis. Coagulation proteins activated by their specific receptors on the surface of VECs subsequently activate these cells, leading to the expression of genes involved in processes such as coagulation, angiogenesis, leucocyte adhesion, and the regulation of the vascular wall tone [31, 32]. When the vascular endothelium is injured, this injury then leads to a disorder of the regional blood flow and the activation of blood platelets and coagulation factors [33].

Coagulation is the process in which a series of plasma coagulation factors are activated [34]. According to differences in the activation pathway and coagulation factors, coagulation is generally divided into two different factor X activation pathways, the intrinsic coagulation pathway and the extrinsic coagulation pathway [35, 36]. When the body is subjected to serious trauma, the traumatized tissue can release a large number of tissue factors (TFs). TFs initiate the extrinsic coagulation pathway [37]. Low-temperature seawater immersion can aggravate local tissue damage, increase the deficiency in the effective circulating blood volume, and cause VEC injury [38, 39]. Therefore, both the intrinsic and extrinsic coagulation pathways could be initiated when the body is exposed to serious trauma during seawater immersion.

In this study, to exclude the effects on the coagulation system caused by the primary injury, the open abdominal injury rat model was carefully established by incisions strictly along the linea alba. Bleeding was rarely observed during the initial stage. In contrast, bleeding could be observed from the edge of the incision in most of the rats in the SW group after 2 hours of seawater immersion, suggesting a haemorrhagic tendency during the later stage in the rats in the SW group. Compared with the rats in the control group, the blood coagulation data of the rats in the SW group changed in a time-dependent manner, with a prolonged PT and shortened APTT, indicating that the coagulation system was mainly activated by the intrinsic coagulation pathway.

During the process of seawater immersion, the activation of coagulation disorders should occur in two stages in the open abdominal injury animals. In the early stage, hyperosmotic cold seawater induced hypertonic body dehydration and haemodynamic disorder and increased the specific volume of the red cells and platelets [40]. Furthermore, the lack of effective circulating blood volume in the body of the model animals led to ischaemia and hypoxia and aggravated metabolic acidosis. Capillary endothelial cells were extensively damaged, followed by the exposure of collagen and the adhesion and aggregation of platelets [41]. Blood coagulation factors XII and XI were activated. Thus, the intrinsic coagulation pathway was initiated [42].

After the activation of the coagulation system, PT is activated, and FIB is consumed; consequently, the fibrinolytic system is activated. With the extension of exposure time, the effective circulating blood volume in the rats of the SW group was further reduced. Microvascular permeability was increased due to continuous and serious local tissue hypoxia. Because a large number of coagulation factors and platelets were consumed, the coagulation and fibrinolytic systems were decompensated [43]. After 2 hours of seawater immersion, plasma concentrations of FDP and D-dimer in the rats of the SW group began to increase. These results indicated a state of DIC [44, 45].

Various factors participate in the process of coagulation and fibrinolysis. Most of these factors are secreted by or are functionally associated with VECs (Figure 2(a)). TF is the receptor for factor VII and is a procoagulant. TF is inhibited by TFPI, which is synthesized primarily by VECs under basal conditions and bound to the surface of VECs [46]. In this study, TFPI was elevated in the later period of DIC, indicating the inhibition of the extrinsic pathway. Thrombin can upregulate VEC P-selectin expression through vWF. The majority of vWF is derived from VECs. vWF binds and stabilizes factor VIII and is a cofactor for the binding of platelets to the exposed extracellular matrix in injured vessel walls [47]. In this study, the expression level of vWF RNA was obviously increased in VECs. The expression level of the vWF protein decreased progressively during the entire coagulation and fibrinolysis activation process, which implied that vWF was consumed dramatically during the process of platelet aggregation.

The fibrinolytic system is activated by the generation of plasmin, which degrades fibrin [48]. Plasmin is formed by the conversion of Plg by at least two types of activators: tPA and uPA [49]. VECs are the principal source of tPA. Plg can be inactivated by PAI-1, which forms stable complexes with either tPA or uPA [50, 51]. In the seawater exposure model, coagulation activation was preceded by the rapid activation of fibrinolysis, reflected by a decrease in the PAI-1 levels and an increase in the Plg and tPA levels.

In conclusion, gene expression profiling technology was applied in this study to analyse the relationship between the injury of VECs and the progression of trauma complicated by seawater immersion. The combination of the hypersaline environment, hyperosmosis, and hypothermia induced by seawater immersion may contribute to the abnormal changes in the morphology and gene expression of VECs, causing a disorder of blood coagulation and DIC. Understanding the molecular mechanism of VEC injury by seawater immersion is helpful for characterizing the course of trauma during seawater immersion and provides a basis for rational and targeted treatments.

## Figures and Tables

**Table 1:** Category of differentially expressed genes in the VECs of rats in the SW group according to the PCR array.

Category	Differentially expressed gene (*A*)		No change gene (*B*)	*A*/(*A* + *B*)
	Upregulated	Downregulated		
*Permeability and vessel tone*	**11**	**1**	**0**	**1.00**
Angiotensin system	Agt, Agtr1	Blr1		1.00
NO system	Nos2, Nos3			1.00
Prostacyclin system	Ptgis			1.00
Endothelin system	Ednra			1.00
Oxidoreductase activity	Nos2, Nos3, Sod1, Xdh			1.00
Regulation of blood pressure	Agtr1, Edn1, Edn2, Ednra, Npr1			1.00
Regulation of vasoconstriction	Edn1, Edn2			1.00
*Angiogenesis*	**14**	**0**	**1**	**0.93**
Negative regulation of angiogenesis	Cxcl4, Plg, Thbs1			1.00
Positive regulation of angiogenesis	Rhob			1.00
Other genes involved in angiogenesis	Angpt1, Col18a1, Fgf1, Fgf2, Flt1, Itgav, Pgf, Serpine1, Tek, Vegfa		Kdr	0.91
*Endothelial cell activation*	**49**	**12**	**15**	**0.80**
Adhesion molecules	Cdh5, Col18a1, Cx3cl1, Fn1, Itga5, Itgav, Itgb1, Rhob, Selp, Tek, Thbs1	Ocln, Sell	Icam1, Itgb3, Pecam, Sele, Vcam1	0.72
Extracellular matrix molecules	Ace, Agt, Angpt1, Cdh5, Col18a1, Cx3cl1, Cxcl1, Cxcl2, Edn1, Edn2, Flt1, Fn1, Ifnb1, Il11, Itga5, Itgb1, Mmp1a, Mmp2, Nppb, Npr1, Pdgfra, Cxcl4, Pgf, Plat, Plau, Plg, Ptgis, Selp, Serpine1, Tek, Tfpi, Thbd, Thbs1, Timp1, Tnfsf10, Vegfa, Vwf	Csf2, Sell, Mmp9, Il1b, Ccl5, Ocln, Casp1, Il7, Tnfsf6	Adam17, Ccl2, Cpb2,Il3, Il6, Kdr, Pecam, Sele, Tgfb1, Vcam1	0.82
Cytokine activity	Cx3cl1, Cxcl1, Cxcl2, Ifnb1, Il11, Cxcl4, Tnfsf10	Csf2, Tnf, Tnfsf6, Il1b, Ccl5, Il7	Ccl2, Il3, Il6	0.81
Thrombin activity	Anxa5, Ecgf1, Plat, Plau, Plg, Serpine1			1.00
Vascular endothelial growth factor Receptor activity	Angpt1, Flt1, Pdgfra		Kdr	0.75
Other genes involved in cell growth	Bax, Col18a1, Cxcl1, Ednra, Fgf1, Il11, Itgav, Itgb1, Nos2, Cxcl4, Pgf, Rhob, Tek, Vegfa, Xdh	Csf2, Blr1, Il1b, Il7	Casp3, Il3, Il6, Itgb3, Kit, Pecam, Tgfb1	0.73
*Endothelial cell injury*	**18**	**8**	**13**	**0.67**
Response to stress	Bax, Bcl2l1, Cx3cl1, Cxcl1, Cxcl2, Cxcl4, Fn1, Tnfrsf6, Ifnb1, Nos2, Selp, Sod1, Tnfsf10	Csf2, Il1b, Ccl5, Casp1, Il7, Tnf, Tnfsf6	Casp3, Ccl2, Il3, Il6, Kit, Pecam, Tgfb1	0.74
Antiapoptosis	Bcl2l1, Vegfa	Birc1b	Bcl2	0.75
Caspase activation	Bax	Casp1	Casp3, Casp6, Cflar	0.40
Induction of apoptosis	Bax, Plg, Tnfrsf6	Casp1	Casp3	0.80
Other apoptosis genes	Bcl2l1, Col18a1, Rhob, Tnfsf10	Tnfsf6	Cradd, Il6, Ripk1, Tnfaip3	0.56
